# Forensic age estimation in males by MRI based on the medial epiphysis of the clavicle

**DOI:** 10.1007/s00414-022-02924-9

**Published:** 2022-12-19

**Authors:** Thomas Widek, Jannick De Tobel, Thomas Ehammer, Pia Genet

**Affiliations:** 1 Diagnostic and Research Institute of Forensic Medicine, Medical University of Graz, Graz, Austria; 2 BioTechMed, Graz, Austria; 3 Department of Diagnostic Sciences - Radiology, Ghent University, Ghent, Belgium; 4 Department of Oral and Maxillofacial Surgery, Geneva University Hospital, Geneva, Switzerland; 5 Graz, Austria; 6 University Centre of Legal Medicine Lausanne, Lausanne University Hospital, Lausanne, Switzerland; 7 University Centre of Legal Medicine Geneva, Geneva University Hospital, Geneva, Switzerland

**Keywords:** Forensic age estimation, Clavicle, Magnetic resonance imaging, Adolescent, Adult

## Abstract

Increasing cross-border migration has brought forensic age assessment into focus in recent decades. Forensic age estimation is based on the three pillars: physical and medical constitution, bone age, and tooth age. Part of the bone age examination includes the assessment of the medial end of the clavicles when the hand bones are already fully developed and a minority must be excluded. Recent research has brought MRI to the forefront as a radiation-free alternative for age assessment. However, there exits only a few studies with large sample size regarding the clavicles and with controversies about staging, motion artifacts, and exclusion based on anatomic norm variants. In the current prospective study, 338 central European male individuals between 13 and 24 years of age underwent MRI examination of the sternoclavicular region. Development was assessed by three blinded raters according to the staging system described by Schmeling et al. and Kellinghaus et al. and related to age by descriptive statistics and transition analyses with a cumulative probit model. In addition, reliability calculations were performed. No statistically significant developmental difference was found between the left and right clavicles. Inter-rater agreement was only moderate, but intra-rater agreement, on the other hand, was good. Stage 3c had a minimum age of 19.36 years and appears to be a good indicator of proof of majority. The minimum age of stage 4 was lower compared with other studies, 20.18 years, and therefore seems not to be an indicator of age of 21 years. In conclusion, we confirmed the value of clavicular MRI in the age estimation process. The transition analysis model is a good approach to circumvent the problems of age mimicry and samples that are not fully equilibrated. Given the moderate agreement between raters, a consensus reading is recommended.

## Introduction

Forensic age assessment in the living based on an X-ray or CT of the epiphysis of the medial end of the clavicles is required when the hand bone development is complete, and is particularly relevant for the age limit of 18 years. The examination is part of a multifactorial medical age assessment process conducted primarily for civil and criminal justice purposes. It includes, according to the recommendations of the Study Group on Forensic Age Diagnostics (AGFAD), a physical examination, an X-ray of the left hand, a dental examination including an X-ray examination of the teeth, and an X-ray or CT examination of the medial end of the clavicles [1]. According to a study by Wittschieber et al. [2], projection radiography examinations are no longer recommended for the evaluation of the sternoclavicular joints because CT is more accurate. However, the associated radiation exposure particular of the CT examinations is subject of controversial discussions due to the lack of a medical indication. The quest for radiation-free alternatives, such as MRI or ultrasound, is therefore a focus of forensic age estimation research in the last decade. Schultz et al. [3, 4] showed that ultrasound examinations might be a good alternative, but they are highly user dependent and there are technical and morphological limitations for age estimations [5, 6]. MRI seems to be the better option due to the possibility for standardization and adequate documentation [7]. Previous studies on using clavicle MRI for age estimation vary in study design and sample size [7–15]. The purpose of this study was to investigate the controversies considering clavicle MRI for age estimation, and to provide data from a large male central European sample, adding to the scarcely available data reported in literature.

## Methods

**Fig. 1:**
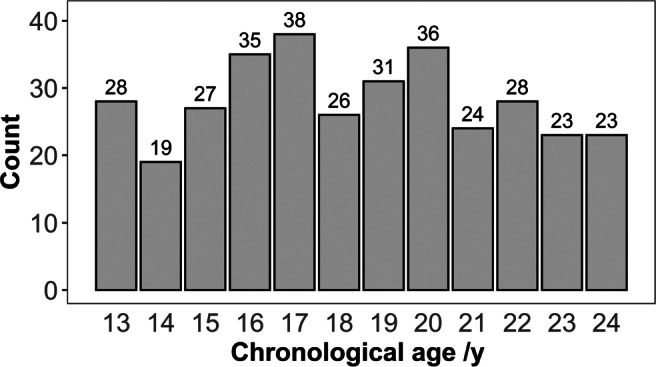
Age distribution of all study subjects in 1-year age groups

A total of 338 male volunteers between 13 and 24 years (range: 13.01–24.98 years, median: 18.90 years, mean: 18.93 years, standard deviation: 3.28 years) participated in this prospective study. All participants were young Caucasian, central European healthy men with documented birth date. Inclusion criteria were age from 13.0 to 25.0 years, informed consent, no medical history of developmental disorders (anamnestically raised), and no severe underweight. Exclusion criteria included MRI contraindications and non-compliance during the examination. The age distribution of the study subjects can be seen in Fig. 1.

All subjects underwent an MRI of both clavicles and the scans were performed at three sites (University Hospital Graz, MRI-Lab Graz University of Technology, MRI Center Privat Hospital of the Sisters of Mercy of the Holy Cross Graz) with two types of 3T MR scanners (MAGNETOM Trio, a TIM system & MAGNETOM Skyra, SIEMENS Healthcare, Erlangen, Germany).

The clavicles were examined in supine position using the standard neck and the standard body matrix coil (SIEMENS Healthcare, Erlangen, Germany). Two sequences inclined parallel to the sternal bone were used. A T2w 2D TSE, with a bigger FOV to image the whole clavicles and a T1w 3D VIBE FS which focused on the sterno-clavicular joints only. The T2w sequence was mainly used to detect pathologies and variants and contributed little to the evaluation. The VIBE sequence was the main sequence used for evaluation. A similar T1w sequence had also been used by other research groups [7, 10] who investigated the clavicles. The total acquisition time was about 11 min. The sequence parameters can be found in Table 1.

**Table 1:** MRI sequence parameter

Sequence	FOV (mm)	Readout matrix (px)	SLT (mm)	TR (ms)	TE (ms)	FA (^∘^)	Acquisition time (min)
T2w 2D TSE	250	256	2.0	2910	65	150^1^	5:57
T1w 3D VIBE FS	170	192	0.9	9.41–9.77	3.69-3.72	12	≈ 5:00

*FOV* field-of-view, *SLT* slice thickness, *TR* repetition time, *TE* echo time, *FA* flip angle

^1^ Refocussing

**Fig. 2:**
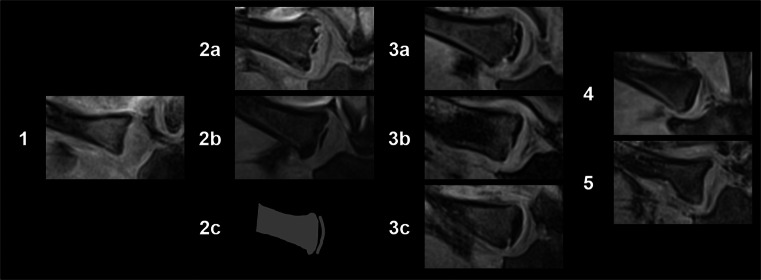
Representative MR images (T1w 3D VIBE FS) of the clavicle stages. Stage 2c was not found in the current study; therefore, a sketch is presented

For the evaluation of the MR clavicle data open-source DICOM viewers (OsiriX 4.1, https://www.osirix-viewer.com and Horos 3.3.5, https://horosproject.org) were used. The data were evaluated by three blinded raters with more than 10-year MRI experience. All three raters used the classification scheme of Schmeling et al. [16] and the sub-classification introduced by Kellinghaus et al. [17] (Table 2). Figure 2 shows representative MR images for the stages. The evaluation was performed on the acquired coronal slices and on multi-planar reformations of the VIBE sequence. The final stage per side was determined by a majority vote. For 91 clavicles (≈ 13%), all three raters assigned a different stage; in these cases, the third rater made the decision by reevaluating the cases knowing the assignments of all three raters.

**Table 2:** Written explanation for the clavicle stages as described by [17, 18]

Stage		Description
1		Ossification centre not ossified
2		Ossification centre ossified, the epiphyseal cartilage not ossified
	2a	The lengthwise epiphyseal measurement is one-third or less compared to the widthwise measurement of the metaphyseal ending
	2b	The lengthwise epiphyseal measurement is over one-third until two-thirds compared to the widthwise measurement of the metaphyseal ending
	2c	The lengthwise epiphyseal measurement is over two-thirds compared to the widthwise measurement of the metaphyseal ending
3		Epiphyseal cartilage is partly ossified
	3a	The epiphyseal-metaphyseal fusion completes one-third or less of the former gap between epiphysis and metaphysis
	3b	The epiphyseal-metaphyseal fusion completes over one-third until two-thirds of the former gap between epiphysis and metaphysis
	3c	The epiphyseal-metaphyseal fusion completes over two-thirds of the former gap between epiphysis and metaphysis
4		Epiphyseal cartilage fully ossified, epiphyseal scar visible
5		Epiphyseal cartilage fully ossified, epiphyseal scar no longer visible

In a first statistical analysis, descriptive statistics were calculated for the individual stages and box-plots were made separately for the two sides. A possible statistical difference between the right and left side was evaluated using a paired Wilcoxon test. For the inter-rater agreement, weighted and unweighted Fleiss’ Kappa and Cohen’s Kappa were calculated. Intra-rater agreement was calculated separately for two raters who re-evaluated fifty randomly selected cases.

Additionally, transition analysis using a cumulative probit model was performed. Point prediction of age and prediction intervals were calculated individually per stage [19–21]. Cross-validation and test-set validation were performed with different test constellations (Table 3); the accuracy of the age prediction was shown by calculating the absolute mean error (chronological age minus point prediction) and the RMSE; only cases with both sides staged were used. Because some stages were underrepresented or not found in the evaluation, stages were combined, resulting in the following stage groups: 1, 2(abc), 3a, 3b, 3c, and 4/5. The best model was then selected to present detailed results and diagrams. For this detailed results, all assessed clavicles were included in a new calculation with the best model approach. The fit of this model was tested with a Lagrange multiplier test and also Cragg and Uhlers pseudo-**R**^2^; was calculated [21]. The ability of the model to differentiate between adults and minors was evaluated by computing the accuracy (percentage of correctly classified individuals), the specificity (percentage of correctly classified minors), and the sensitivity (percentage of correctly classified adults) with respect to the point prediction [8]. Finally, normed likelihood curves were plotted for the collapsed stages [20, 22]. All statistical analyses were done using the software R v4.1.0 [23] including the R-packages “irr” [24], “irrCAC” [25], “pscl” [26], “MASS” [27], “VGAM” [28], and with modified R-scripts provided by Lyle Konigsberg [29].

**Table 3:** Cross-validation and test-set constellations

Model training dataset	Count	Cross-validation fold	Test set
Clavicula right	288	10	Clavicula right
Clavicula left	288	10	Clavicula left
Whole set^1^	576	10	Whole set
Clavicula right	288	—	Clavicula left
Clavicula left	288	—	Clavicula right
Half set max^2^	288	10	Half set max
Half set min^2^	288	10	Half set min

^1^ Whole set: merged dataset of right and left clavicula stages

^2^ Half set max/min: maximal/minimal stage of both sides was taken

The local ethics committee granted ethical clearance for the study. All participants gave written informed consent prior to study participation, with consent given by legal guardians for minors.

## Results

**Fig. 3:**
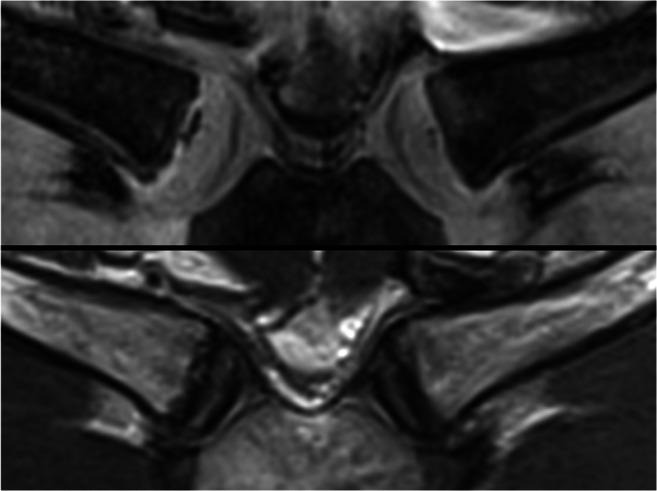
MR images of a 17.74-year-old male with stage 3a (right) and 2a (left) according to the enhanced Kellinghaus classification. Top: T1w 3D VIBE. Bottom: T2w TSE

Data of 338 male subjects were evaluated. Some cases respectively single clavicles had to be excluded due to motion artefacts (3%) or anatomical shape variants (e.g., “fish-mouth-like” depression, 8%). Finally, of 314 subjects, 602 (R: 304, L: 298) clavicles could be included in the statistical analysis. Figure 3 shows one example for the used sequences in a 17.74-year-old male individual.

**Fig. 4:**
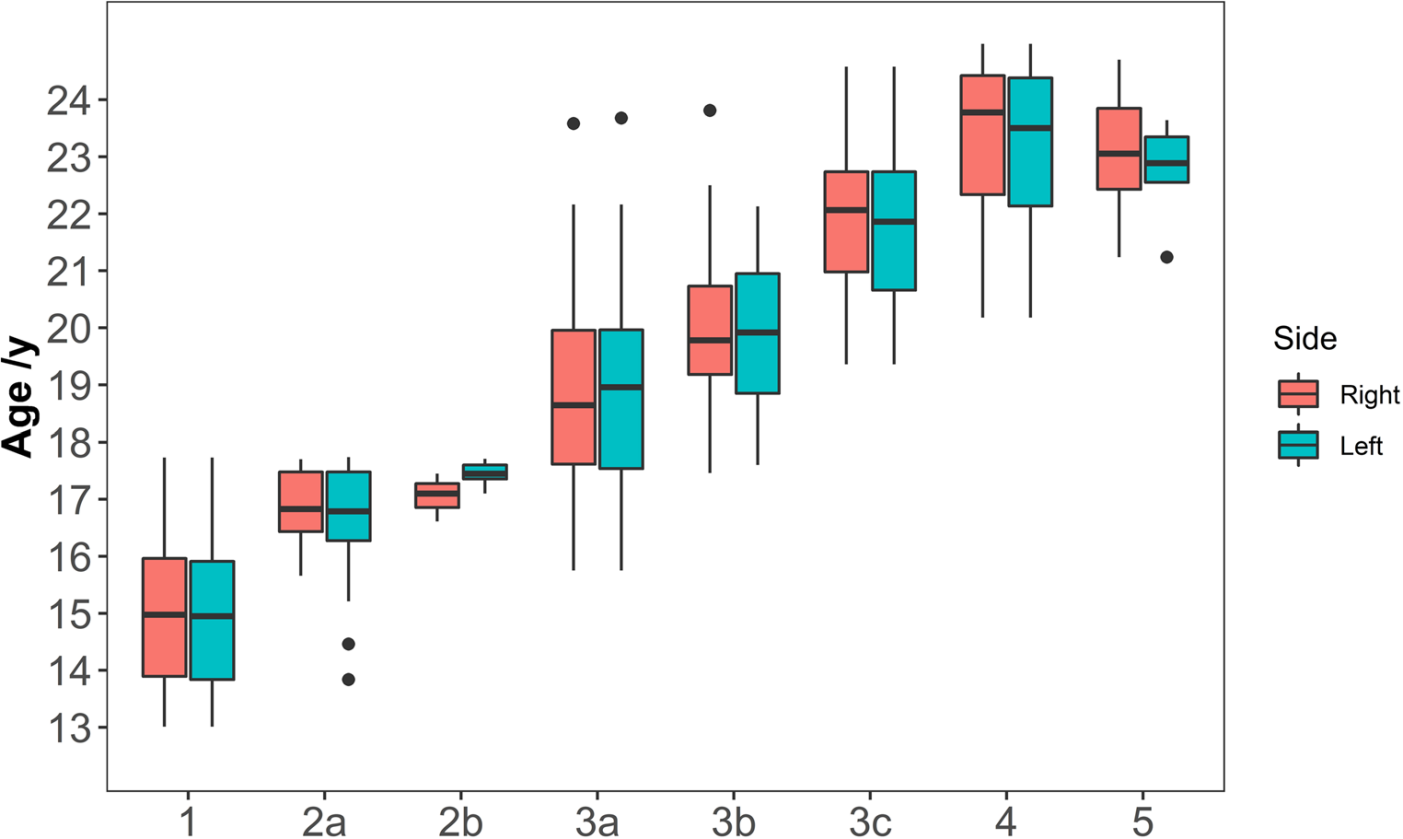
Box and whisker plot of the clavicle ossification. The *x*-axis shows the stages, and the *y*-axis displays the chronological age. The boxes cover the lower and upper quartile and the thick transverse line displays the median. The maximum and minimum values are shown by the whisker ends and outliers are indicated by a black dot. Outliers are defined as values which lie outside 1.5 times the interquartile range (IQR) of the lower or upper quartile, respectively. Note, that stage 2c was not found in the current sample

The descriptive statistics including mean, standard deviation, minimum, maximum, median, and lower and upper quartile of the individual stages found are displayed in Table 4, separately for the right and the left side. Figure 4 shows the corresponding boxplots of the left and right clavicle staging results.

**Table 4:** Descriptive statistical data (in years) for the individual ossification stages of the medial end of the clavicle (separated for the right and the left side)

Stage	Side	*N*	Mean	SD	Min	LQ	Median	UQ	Max
1	Right	86	15.04	1.29	13.01	13.89	14.97	15.96	17.73
	Left	81	14.98	1.31	13.01	13.84	14.95	15.91	17.73
2a	Right	12	16.92	0.63	15.66	16.44	16.83	17.48	17.70
	Left	16	16.58	1.18	13.84	16.28	16.79	17.48	17.74
2b	Right	3	17.05	0.42	16.61	16.86	17.10	17.27	17.45
	Left	7	17.45	0.21	17.10	17.36	17.45	17.60	17.71
2c	−	0	−	−	−	−	−	−	−
3a	Right	88	18.82	1.57	15.75	17.62	18.64	19.95	23.58
	Left	78	18.84	1.54	15.75	17.54	18.96	19.96	23.68
3b	Right	23	20.14	1.47	17.46	19.18	19.78	20.73	23.81
	Left	17	19.89	1.36	17.6	18.85	19.92	20.95	22.13
3c	Right	44	21.96	1.27	19.36	20.98	22.06	22.73	24.58
	Left	48	21.82	1.42	19.36	20.66	21.86	22.73	24.58
4	Right	38	23.33	1.41	20.18	22.34	23.78	24.42	24.98
	Left	46	23.16	1.37	20.18	22.13	23.51	24.38	24.98
5	Right	10	23.01	1.11	21.24	22.43	23.05	23.85	24.70
	Left	5	22.73	0.93	21.24	22.55	22.89	23.35	23.64

*SD* standard deviation, *LQ* lower quartile, *UQ* upper quartile

In 288 cases, both sides were evaluated. Of these cases, about 80% (*n*= 230) of the subjects showed the same stage for the right and left side. The remaining 58 cases showed a difference of one stage (*n*= 47) or a difference of two or more stages (*n*= 11). An applied paired Wilcoxon test showed no significant results (significance level *p*< 0.05), meaning there was no tendency for the left or right side for an accelerated or retarded development.

**Fig. 5:**
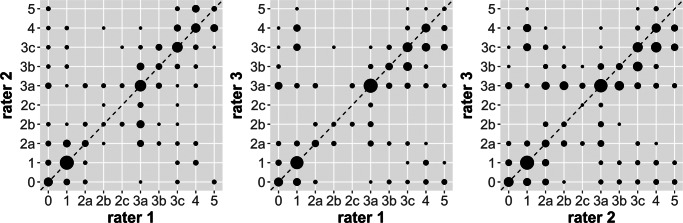
Graphical representation of the inter-rater agreement of two reviewers each. The *x* and *y* axes show the different clavicle stages. Zero stands for not evaluable. Dots on the identity line (dashed line) represent complete agreement. The size of the dots indicates the frequency of occurrence

Inter-rater agreement of the three raters showed only a moderate [30] Fleiss’ Kappa with *κ*_*f*_ = 0.43 (*p* < 0.05), including all clavicles (*n*= 676) independent of their evaluability. A linearly weighted Fleiss’ Kappa, considering only clavicles (*n*= 551) staged by all three raters, yielded a substantial agreement with *κ*_*fw*_ = 0.65. An additionally calculated Krippendorff’s Alpha resulted in a value of *α* = 0.647 which means insufficient agreement [31]. A graphical display of the agreement can be seen in Fig. 5.

The individual agreement (only staged clavicles) between two raters was calculated with weighted Cohen’s Kappa and varied between moderate and good (Table 5).

**Table 5:** Inter-rater agreement between the individual raters

	*N*	*κ*_*w*_	*p*-value
R1R2	573	0.54	< 0.05
R1R3	582	0.75	< 0.05
R2R3	561	0.64	< 0.05

*R* rater, *N* count, *κ*_*w*_ weighted Kappa

Intra-rater agreements were calculated separately for two raters and showed moderate to good results with an unweighted Cohen’s Kappa, when including all re-evaluated cases. Excluding the cases in which one of the two evaluations was classified as not evaluable, the now applicable weighted Kappa showed good to very good values (Table 6).

**Table 6:** Intra-rater agreement for two raters

	Unweighted Cohen’s Kappa			Weighted Cohen’s Kappa		
	*N*	*κ*	*p*-value	*N*	*κ*_*w*_	*p*-value
R2	100	0.60	< 0.05	92	0.74	< 0.05
R3	100	0.70	< 0.05	91	0.91	< 0.05

*R* rater, *N* count, *κ*_*w*_ weighted Kappa

For the transition analysis (TA) models, different approaches (Table 3) were used, and the results of the cross-validation can be seen in Table 7. In the TA, the lowest and the highest stage have method-related no point prediction, so these values were set to the minimum and maximum value of the sample, respectively, for the calculation.

**Table 7:** Cross-validated performance of different model approaches (in years)

Model	Test set	MAE	RMSE
Clavicula right	Clavicula right	1.50	1.86
Clavicula left	Clavicula left	1.53	1.90
Whole set^1^	Whole set	1.51	1.88
Clavicula right	Clavicula left	1.53	1.91
Clavicula left	Clavicula right	1.50	1.88
Half set max^2^	Half set max	1.49	1.84
Half set min^2^	Half set min	1.52	1.91

^1^ Whole set: merged dataset of right and left clavicula stages

^2^ Half set max/min: maximal/minimal stage of both sides was taken

*MAE* mean absolute error, *RMSE* root mean square error

**Fig. 6:**
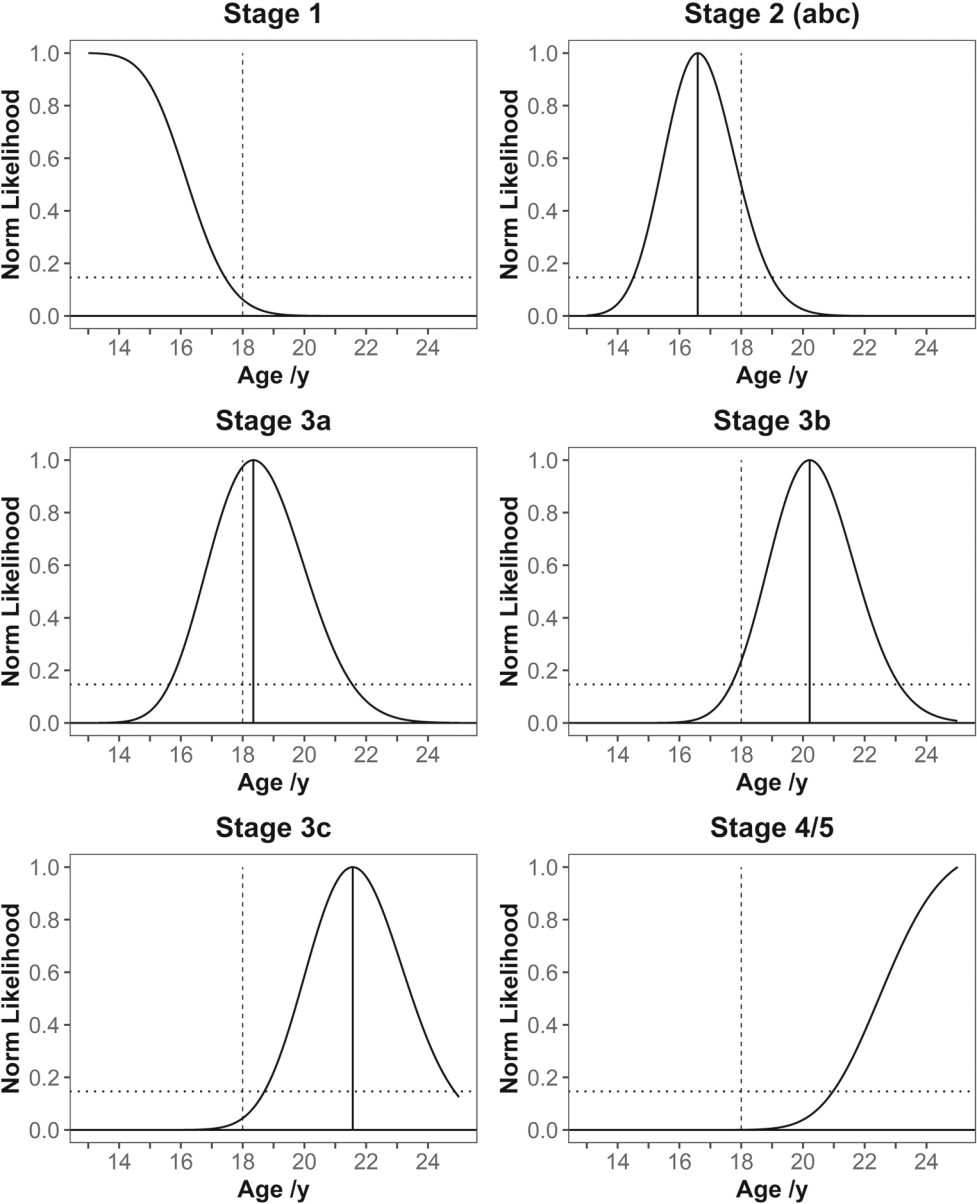
Normed maximum likelihood curves (max. stages model). The solid vertical line represents the point prediction, and the dashed vertical line displays the age of 18. The horizontal line shows the likelihood of 0.1465 indicating the 95% prediction interval

Although no model appears to outperform the other, after cross-validation, the nominally best model (“Half set max”) based on MAE and RSME was selected for more detailed investigation. For this purpose, all data where at least one side was staged were included. The highest reached stage (max. stage) was chosen resulting in 314 cases. The results can be seen in Table 8a, b and in Fig. 6.

**Table 8:** Extended results from the evaluation of the model using the highest achieved stages of the two clavicles

(a)		(b)		
		Stage	MLA	95% prediction interval TA
*N*	314	1	–	13.00–17.43
MAE	1.482 y	2 (abc)	16.59	14.50–18.99
RMSE	1.852 y	3a	18.35	15.62–21.55
LM test	*p* = 0.948	3b	20.22	17.68–23.11
$R_{\mathrm {Cragg \& Uhlers}}^{2}$	0.839	3c	21.56	18.70–24.86
Accuracy	88.5%	4/5	–	20.97–25.00
Specificiy	73.7%			
Sensitivity	100%			

Note that the lower bound of the prediction interval in the lowest stage reflects the minimum age in the study sample. Similarly, the upper bound in the highest stage corresponds to the maximum age in the study sample

*MAE* mean absolute error

*RMSE* root mean square error

*LM* test Lagrange multiplier test

*MLA* point prediction (in years)

*TA* transition analysis

## Discussion

The examination of the medial end of the clavicles for the purpose of forensic age estimation in the living is currently carried out using ionizing radiation with either CT or more rarely plain X-ray [32]. Recently, a few groups have started to use MRI as an alternative modality [7–11, 14, 15]. The current study also used MRI and a 5-stage classification with subdivisions of stages 2 and 3 according to Schmeling and Kellinghaus [16, 17]. About 8% of the clavicles had to be excluded due to anatomical shape variants and about 3% due to motion artefacts. The total exclusions were similar to the study of Schmidt et al. [7]. The number of exclusions due to shape variants seems to be highly dependent on the raters. There was a high variance between different studies, where the numbers vary between 2.1% [8] and 21.3% [15], but there was also a high variance in the results of the individual raters in the current study. Motion-related exclusions were similar to the studies of Hillewig and De Tobel [8, 11] but only half compared to Vieth et al. [15]. Since these three studies positioned the subjects in prone position and the current study used the supine position, there appears to be no advantage to using either position.

Staging clavicle development appears to be more difficult than other regions used for age estimation. This can be seen both in the comparison of the three raters and in the direct individual comparison of two raters. The results are similar to the study by De Tobel et al. [8] and their proposal to evaluate the clavicles in consensus of at least two raters is also one of the conclusions of this study. It is worth mentioning that in the current study, one of the raters was from a different institute and that the agreement between raters from different institutes was lower than between raters from the same institute. Therefore, it appears that there is also additional an institute-dependent behavior in the evaluation of MR clavicle images. This is also insisted by the good intra-rater agreement. One solution for a more objective and reliable evaluation could be an automatic evaluation using deep learning algorithms, as shown in the publications of Stern et al. [33, 34].

Similar to other MR studies [8, 11, 15], stage 2a and 2b were found rarely and stage 2c was not detected at all. Also in the CT study by Wittschieber et al. [35], the number of individuals with stage 2 was low. One reason might be that stage 2 and its subdivisions are quick transition stages which are seldom found, and single bone bridges build very early in the ossification process. Another reason might be that current MR sequences and equipment are able to detect bridges between metaphyse and epiphyse earlier and therefore it comes to an upstaging. However, this could not be confirmed in smaller postmortem comparative studies [36, 37] between MR and CT, but studies with a larger sample are necessary. Nevertheless the evaluation of small bone bridges seems to be more difficult in MR images than in CT images according to the current study’s raters. This might be due to lower resolution, possible blurring, and other artifacts in the MR data. This might also be one explanation for the lower agreement between the raters.

Similar to the study done by De Tobel et al.[8], the substages 2a, b, and c were merged to stage 2 in the transition analysis. They also point out that the subdivision of stage 2 on MR images is not necessary [8], which also seems plausible according to the results of this study. However, the number of stage 2 cases in this study was too small to make a definitive recommendation. On the other hand, the suggestion by Wittschieber et al. [38] to subdivide stage 3a according to the width of epiphyseal ossification seems promising. This was also discussed in an MRI study by De Tobel et al. [8]. However, both studies examined only a small study population, so studies with a larger sample are needed.

Stage 5 was found in total only 15 times for the left and right clavicle. This is in concordance with other studies [7, 12] which found similar numbers or no stage 5 at all. One reason could be the upper age limit of the sample, as already mentioned by Schmidt et al. [7]. Another reason might be the long visibility of the epiphyseal scar in the used MR sequences. Hillewig et al. [11] reported difficulties in distinguishing between stages 4 and 5, and therefore decided to use a four stage system. However, as also stated by Schmidt et al. [7], stage 5 might be helpful in certain legal circumstances as the minimum age of stage 5 in the current study was over 21 years of age. Notable is the fact that in the current study, the mean age of stage 5 was lower than for stage 4. However, as stage 5 is a terminal stage, this should not be overrated. For the transition analysis, stages 4 and 5 were merged due to the low numbers of stage 5.

Both Hillewig and De Tobel [8, 11] reported difficulties in distinguishing stage 1 from stage 4/5. Schmidt et al. [7] contradicted this by pointing out that the shape of the medial clavicle ends, as described in [12], clearly allows a distinction. In the current study, there were also individual cases in which it was difficult to make a clear distinction and the evaluators’ classifications differed widely. In these cases, it is helpful to consider the thickness of the clavicular cartilage and the shape and structure of the surrounding bones, such as the manubrium or the first rib, since there are also age-related changes, as noted by Martínez Vera et al. [39] for the manubrium.

The comparison of the descriptive stage values of this study with the study done by Schmidt et al. [7] who investigated a similar sample with a comparable MR sequence showed overall slightly younger mean values with lower values for the standard deviation in the current study. The minimum values differed for the stages 2b, 3a, and 4 up to ± 1,5 years. In the current study, the minimum age for stage 3a was about 1 year lower. This is perhaps due to a different perception of narrow bone bridges. The minimum age for stage 4 was 20.18 years, surprisingly much lower than in the comparative study [7], where the minimum age for males was 21.7 years. A minimum age for stage 4 below 21 years was also not found in comparable radiographic studies [11, 18, 35]. For the maxima, the difference was most interesting in the stage 2 cases, as in the current study, the values for both stage 2a and stage 2b were below 18. However, this could again be due to the perception of narrow bone bridges.

Different constellations were used for the transition analysis, but the different models did not show much difference in their performance. However, the model using the highest stage found in both medial clavicle ends was marginally better than the rest and more detailed results were calculated only for this constellation. The selection of the highest stage if the two clavicles showed different stages was also applied in other studies [7, 18]. A Lagrange multiplier test showed the good fitting of the probit model to the data. Accuracy for minor and adult determination was over 88% in terms of point prediction (MLA), and no adult was classified as a minor. However, about one-quarter of the minors were identified as adults when looking only at the MLA. Compared to the results of a forward continuation transition analysis approach [8], the results are slightly better. Nevertheless, miss-classification of minors should always be avoided. Therefore, relying on the MLA alone is inappropriate, and a prediction interval should always be used. When using the lower bound of the prediction interval, 100% of the minors are classified as minors. On contrary, the accuracy and the classification of the adults are consequently decreased.

Combining both the right and left clavicles after estimating the transition analysis parameters was not done, as over 80% of the clavicles showed the same stage and no significant difference was found in the applied paired Wilcoxon test.

There are some limitations in this study that need to be discussed. First, socioeconomic status was not documented and therefore could not be considered in the evaluation. However, since all study participants resided in a country with a high socioeconomic level, no influence was expected. Second, the sample consisted of males only. This was decided for financial reasons in view of a larger sample. After all, the vast majority of cases in forensic age estimation are male.

## Conclusion

In conclusion, MRI and a transition analysis with a cumulative probit approach were shown to be applicable for forensic age estimation based on the medial end of the clavicle. However, the staging is more demanding than in other anatomical regions and a consensus reading of at least two raters is recommended. Transition analysis seems to be a good tool to reliably classify minors when prediction intervals are used, but the high number of misclassified adults indicates that the prediction intervals are too wide. Therefore, multi-factorial age assessment is recommended as it will narrow the prediction intervals. In addition, future studies should include more older subjects, to obtain a better differentiation and understanding of stages 4 and 5.
